# Alveolar Type II Cells or Mesenchymal Stem Cells: Comparison of Two Different Cell Therapies for the Treatment of Acute Lung Injury in Rats

**DOI:** 10.3390/cells9081816

**Published:** 2020-07-31

**Authors:** Raquel Guillamat-Prats, Marta Camprubí-Rimblas, Ferranda Puig, Raquel Herrero, Neus Tantinyà, Anna Serrano-Mollar, Antonio Artigas

**Affiliations:** 1 Centro de Investigaciones Biomédicas en Red de Enfermedades Respiratorias, CIBERES-Instituto De Salud Carlos III, 28029 Madrid, Spain; mcamprubi@tauli.cat (M.C.-R.); ferranda@gmail.com (F.P.); raquelher@hotmail.com (R.H.); neus.tantinya@gmail.com (N.T.); anna.serranomollar@iibb.csic.es (A.S.-M.); aartigas@tauli.cat (A.A.); 2 Institut d’Investigació i Innovació Parc Taulí (I3PT); 08208 Sabadell, Spain; 3 Universitat Autònoma de Barcelona, 08193 Bellaterra, Spain; 4 Intensive Care Medicine Service, Hospital Universitario de Getafe, 28905 Getafe, Spain; 5 Department of Experimental Pathology, Institut d’Investigacions Biomèdiques de Barcelona, Consejo Superior de Investigaciones Científicas (IIBB-CSIC), Institut d’Investigacions Biomédiques August Pi i Sunyer (IDIBAPS), 08036 Barcelona, Spain; 6 Critical Care Center, Corporació Sanitària i Universitària Parc Taulí, 08208 Sabadell, Spain

**Keywords:** cell therapy, alveolar type II cells, mesenchymal stem cells, acute lung injury, ARDS, ALI

## Abstract

The use of cell therapies has recently increased for the treatment of pulmonary diseases. Mesenchymal stem/stromal cells (MSCs) and alveolar type II cells (ATII) are the main cell-based therapies used for the treatment of acute respiratory distress syndrome (ARDS). Many pre-clinical studies have shown that both therapies generate positive outcomes; however, the differences in the efficiency of MSCs or ATII for reducing lung damage remains to be studied. We compared the potential of both cell therapies, administering them using the same route and dose and equal time points in a sustained acute lung injury (ALI) model. We found that the MSCs and ATII cells have similar therapeutic effects when we tested them in a hydrochloric acid and lipopolysaccharide (HCl-LPS) two-hit ALI model. Both therapies were able to reduce proinflammatory cytokines, decrease neutrophil infiltration, reduce permeability, and moderate hemorrhage and interstitial edema. Although MSCs and ATII cells have been described as targeting different cellular and molecular mechanisms, our data indicates that both cell therapies are successful for the treatment of ALI, with similar beneficial results. Understanding direct cell crosstalk and the factors released from each cell will open the door to more accurate drugs being able to target specific pathways and offer new curative options for ARDS.

## 1. Introduction

Cell-based therapies have gained interest in the last few years for the treatment of several lung diseases. Multipotent mesenchymal stem/stromal cells (MSCs) have been used for the treatment of lung diseases, and their use has rapidly progressed over the past decade [1,2,3,4,5,6]. MSCs modulate the host immune response, secrete growth factors and cytokines that reduce inflammation [7,8,9,10], and release antimicrobial peptides with bactericidal capacity [11,12,13]. MSCs are immune evasive cells that do not induce rejection, and thus can be used without the need for immunosuppression in patients [14,15,16]. Additionally, MSCs can restore the lungs after damage due to their ability to differentiate into alveolar type II cells (ATII). ATII cells have also been used as a cell therapy in acute and chronic diseases [17,18,19,20,21,22,23], and they share some of the MSCs’ properties, such as their ability to secrete growth factors and cytokines that reduce inflammation and enhance tissue repair [19,20,24]. ATII cells synthesize surfactant and other proteins and lipids with anti-inflammatory and antimicrobial effects [18,24,25]. 

The main risk for the use of cell therapies is the possibility of deregulated proliferation and tumor formation or the indiscriminate migration into other healthy tissues [15,26,27,28]. ATII cells are more differentiated compared to MSCs, and this might reduce both risks. Nevertheless, MSCs have more proliferative capacity, which might enhance their ability to survive long enough in the injury site and increase their beneficial effects [15,29,30]. MSCs cells can be obtained from many adult and embryonic tissues, such as bone marrow, adipose tissue, or umbilical cord blood. MSCs are easier to obtain than ATII, which need to be isolated from healthy death lung donors. Nowadays, ATII can also be obtained by the differentiation of adult pluripotent cells in vitro [31,32,33,34,35]. Both cell types were tested in clinical trials for several lung diseases, with a good safety record in patients [36,37,38,39,40,41].

MSCs and ATII have been used to specifically target acute respiratory distress syndrome in pre-clinical models (ALI) [1,6,14,42] and in the clinics in humans (ARDS) [10,14,19,43,44]. ARDS is a life-threatening disease, with a high mortality rate of approximately 40% and a rapidly progressive condition in critically ill patients, characterized by complex mechanisms and pathologic processes leading to impaired gas exchange with acute hypoxemia [45,46,47,48,49]. The main hallmarks of ARDS are increased alveolar–capillary barrier permeability, an influx of inflammatory cells in the lungs, the increased local expression of proinflammatory cytokines and chemokines, and a local procoagulant state [45,50,51,52]. The restoration of the alveolar damage depends on the equilibrium of proinflammatory/antiinflammatory interactions and the modulation of many molecular pathways [45,51]. There is still no effective pharmacologic therapy for ARDS, and the management treatment options remain supportive. 

MSCs were used to treat lung injury in several animal models, resulting in beneficial effects and positive outcomes in survival, and constitute a promising therapy for ARDS [1,6,7,8,9,10,11,12,13,53]. Additionally, our group and others have tested ATII for the treatment of lung injury, showing a decrease in pro-inflammatory cytokines, the modulation of macrophage activation, and a reduction of edema [19,20,24].

We assume that the mechanisms between MSCs and ATII cells might be different due to their different gene expression profile and their intrinsic nature; however, whether MSCs or ATII are more efficient in reducing ALI or ARDS remains to be compared. In this study, we investigated the effects of both cell therapies in the acute phase following lung injury and compare the efficacy of MSC to the use of ATII cells in tissue injury repair and the regulation of inflammation using a sustained acute pre-clinical lung injury rat model.

## 2. Materials and Methods

### 2.1. Animals

Male Sprague-Dawley rats (Charles River, France) weighing 200–225 g at the beginning of the experiment were used, in accordance with the European Community Directive 86/609/EEC and Spanish guidelines for experimental animals. The study was approved by the institutional committee of Autonomous University of Barcelona and the Animal Experimentation Committee of Generalitat de Catalunya. The animals were housed under a 12:12 h light–dark cycle, and food (A04: Panlab, Barcelona, Spain) and tap water were available ad libitum.

### 2.2. Experimental Groups 

**Figure 1:**
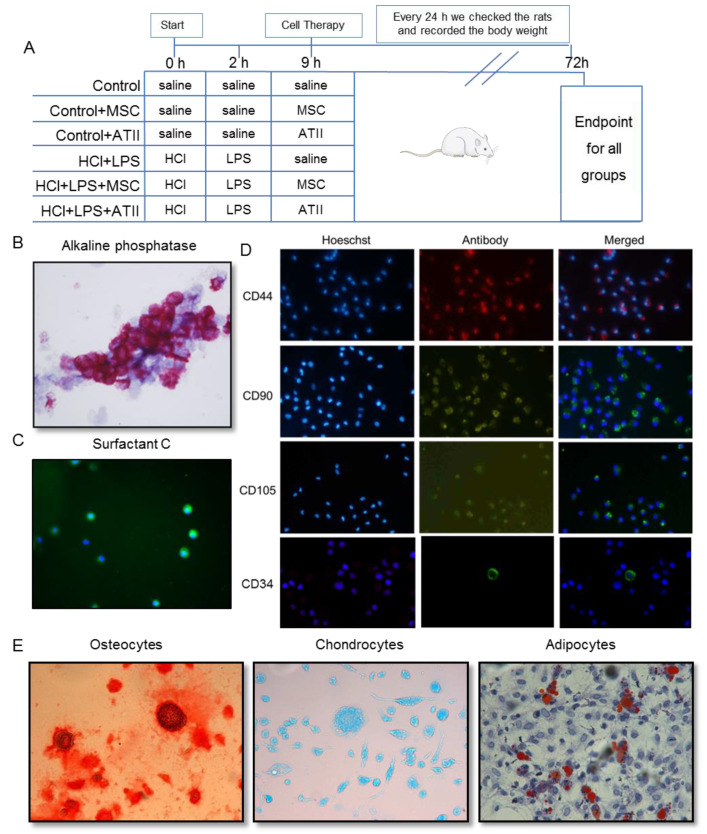
Experimental procedure schema and multipotent mesenchymal stem cell (MSC) and alveolar type II cell (ATII) purity. (**A**) Animal experimental design: rats of 200–225 g body weight at the beginning of the experiment were randomized in six experimental groups as indicated. At 0, 2, and 9 h an intratracheal instillation was administered as indicated; the body weights were recorded every 24 h; and all the animals were sacrificed 72 h after starting the experiment. (**B**) Alveolar type II (ATII) cells stained with alkaline phosphatase (100×). In dark pink, we can identify the positive cells, therefore the real ATII. The purity for the ATII cells was 86 ± 3%. (**C**) Surfactant C staining for alveolar type II cells. Surfactant C (in green, fluorescein isothiocyanate (FITC)) and nuclear staining (in blue, Hoechst 33342) to confirm the ATII cell purity. (**D**) Mesenchymal stem cells stained by CD44 (in red, Texas Red), CD90 (in green, FITC), CD105 (in green, FITC), and CD34 (in green, FITC). Nuclei can be observed in blue by Hoechst 33342 staining. Magnification used is 100×. MSC should express CD44, CD90, and CD105 and should be negative for CD34. (**E**) MSC were differentiated into different lineages. Panel E shows the differentiation to osteocytes stained with Alizarin Red, to chondrocytes stained with Alzian Blue, and to adipocytes stained in oil-red -O staining (200× magnification). The purity of MSC was 78 ± 5%.

The animals were randomly distributed into six experimental groups (Figure 1A): 

(1) Control: saline instillation at 0, 2, and 9 h; 

(2) Control + MSCs: saline instillation at 0 and 2 h followed by MSC instillation after 9 h; 

(3) Control + ATII: saline instillation at 0 and 2 h followed by ATII cell instillation after 9 h; 

(4) HCl + LPS: HCl instillation at 0 h, LPS administration at 2 h, and saline instillation after 9 h;

(5) HCl + LPS + MSCs: HCl + LPS, as in the previous group, plus MSC instillation after 9 h; 

(6) HCl + LPS + ATII: HCl + LPS, as in the previous group, plus ATII cell instillation after 9 h. 

The following section describes the experiments as they were conducted.

### 2.3. HCl and LPS Induced ALI and Cell Infusion

ALI was induced as in our previous study [54]. The animals received an intratracheal instillation of 300 µL of HCl (0.1 M at pH = 1.4), and 2 h later an intratracheal instillation of the endotoxin lipopolysaccharide (LPS; Escherichia coli 055:B5, Sigma, St. Louis, MO, USA, 30 µg/g of body weight) dissolved in 500 µL of saline under sevoflurane anesthesia. The control animals received the same volume of saline. During the experiment, the animal body weights were recorded every day. Nine hours after the endotracheal HCl administration (or saline, in the case of control animals), recipient animals were transplanted with ATII cells or MSCs endotracheally by the trans-oral route under sevoflurane anesthesia. Each animal received a single bolus of 2.5 × 10^6^ cells (ATII or MSCs; after knowing the purity and using the appropriate calculations. we administered 2.5 × 10^6^ pure cells) suspended in 400 μL of sterile saline. The control groups received the same dose of cells. The animals were killed 72 h after the induction of ALI. 

### 2.4. Isolation and Purification of Alveolar Type II Cells

As previously described, fresh ATII cells were isolated from healthy donor animals [17]. Briefly, the lungs were removed and five bronchoalveolar lavages (BAL) with 10 mL of saline were performed. The lungs were digested with 0.25% trypsin (T8003; Sigma, St. Louis, MO, USA) dissolved in saline (60 mL), keeping the lungs submerged in a saline bath at 37 °C for 30 min to keep the temperature stable during the process. The lungs were chopped in 1–2 mm^2^ pieces, treated with 25 mL of DNase (75 U/mL) (Roche Diagnostics, Manheim, Germany), and filtered through 100 and 40 μm nylon meshes. Centrifuge cell separation was performed using Percoll, the enriched cell band population was mixed with DNase solution (20 U/mL) and centrifuged at 500× *g* for 15 min, and the pellet was resuspended in 5 mL of DCCM-1 (Biological Industries, Kibbutz Beit Haemek, Israel) supplemented with 2% L-glutamine (Sigma, St. Louis, MO, USA) and subjected to differential attachment on a plastic Petri dish. No adherent ATII cells were collected after 1 h, and they were counted to establish the final yield of freshly purified cells and administered fresh to the animals. The ATII cell viability was evaluated with trypan blue (Sigma, St. Louis, MO, USA) and its purity by alkaline phosphatase staining (Sigma, St. Louis, MO, USA), and the expression of surfactant C (SPC, Santa Cruz, USA, ref sc-13979, rabbit, 1:100) was measured by immunofluorescence and marked by the secondary anti-rabbit antibody (Santa Cruz, 136 USA, ref. sc2359. FITC, 1:100). SPC is observed in green (FITC) in Figure 1C and the stained nuclei with Hoechst33342 (Life technologies) (Figure 1B,C). The purity of the ATII cells was 86 ± 3%. 

### 2.5. Isolation and Purification of Mesenchymal Stem Cells and Differentiation to Osteocytes, Chondrocytes, and Adipocytes

Femurs were obtained from healthy donor animals. After the removal of the peripheral muscle tissue, the femurs were briefly soaked with alcohol. Bone marrow was isolated by flushing the bones with sterile phosphate-buffered saline (PBS). The bone marrow suspension was filtered with a 100-mesh filter and then centrifuged. The pellets were resuspended in growth medium composed of DMEM (Gibco, Thermo Fisher, Waltham, MA, USA) supplemented with 10% fetal bovine serum (FBS, Thermo Fisher, Waltham, MA, USA), and the cells were plated in T75 flasks followed by incubating at 37 °C and 5% CO2. After 48 h, the media were changed every 3 days until 80–90% confluence. After 1 week, MSCs were detached to the plate and administered to the animals. The purity of the MSCs was tested by their ability to adhere to plastic in standard culture medium and by the expression of CD44 (Abcam, Cambridge, UK, ref. ab24504, rabbit, 1:10), CD90 (Abcam, Cambridge, UK, ref. ab225, mouse, 1:1000), and CD105 (Abcam, Cambridge, UK, ref. ab156756, mouse, 1:100) (Figure 1D) and the lack of CD45 (Abcam, Cambridge, UK, ref. ab10558, rabbit, 1:200) (not shown) and CD34 (Abcam, Cambridge, UK, ref. 81289, rabbit, 1:200), measured by immunofluorescence. The cells were incubated with the primary indicated antibodies individually and revealed with a secondary anti-rabbit antibody (Santa Cruz, USA, ref. sc3917-TRF, 1:200) or anti-rabbit antibody (Santa Cruz, 136 USA, ref. sc2359–FITC, 1:100) and anti-mouse antibody (Santa Cruz, USA, ref. sc516140. FITC, 1:100). CD44 is observed in red (Texas red) and CD90, CD105, and CD34 in green (FITC) in Figure 1D. The nuclei were stained using Hoechst33342 (Life technologies), and we counted at least 500 cells using a fluorescence microscope and calculate the percentage of purity. The purity of MSCs was 78 ± 5%. 

The MSCs’ capacity to differentiate into osteogenic, chondrogenic, and adipogenic lineages was also evaluated [28]. Confluent MSCs were cultured at 37 °C and 5% CO2 with the respective differentiation media: a StemPro^™^ Osteogenesis (Pierce; Thermo Scientific; Rockford, IL, USA, ref. A10072-01), Chondrogenesis (Pierce; Thermo Scientific; Rockford, IL, USA, ref. A10071-01), or Adipogenesis (Pierce; Thermo Scientific; Rockford, IL, USA, ref. A10070-01) Differentiation Kit. The media were changed every 48 h. After 7 days, adipocytes were fixed for 30 min with 10% formalin, washed with deionized water, incubated with 60% isopropanol for 5 min, and incubated in Oil Red O solution for 5 min. The cells were washed with current water, incubated with hematoxylin for 1 min, and rinsed with current water. After 14 days, chondrocytes were fixed for 30 min with 4% formalin, washed with DPBS, stained for 30 min with 1% Alcian Blue, and rinsed with 0.1 N HCl and distilled water. After 21 days, osteocytes were washed with DPBS, fixed for 30 min with 4% formalin, washed with distilled water, stained for 2 min with 2% Alizarin Red, and washed with distilled water. The preparations were mounted and imaged using a Nikon Eclipse Ti microscope (Figure 1E).

### 2.6. Endpoint

The animals were anesthetized intraperitoneally with ketamine (90 mg/kg) and xylazine (10 mg/kg) and were exsanguinated from the abdominal aorta at 72 h. The lungs were removed and weighed. Then, the left hilum was tied with a suture, and the right lung was removed and flash-frozen in liquid nitrogen. The left lung was washed with five separate 5 mL aliquots of 0.9% NaCl containing 1mM EDTA for BAL collection or fixed with 4% paraformaldehyde for histological analysis. 

### 2.7. Bronchoalveolar Lavage Fluid Analysis

The BAL fluid samples were processed, and we counted the total cells. Lymphocytes, polymorphonuclear, and monocytes/macrophages were evaluated and counted in cytospin preparations stained with the diff-quick method (Pancreac Quimica SAU; Spain). The total protein concentration in BAL fluid was determined by the bicinchoninic acid method (BCA) (Pierce; Thermo Scientific; Rockford, IL, USA). The concentration of IgM in BAL fluid was measured using an ELISA (Abcam, Cambridge, UK). The myeloperoxidase (MPO) concentration in BAL fluid was determined with an ELISA assay (Hycult Biotech, Uden, The Netherlands) following the manufacturer’s instructions.

### 2.8. Histological Studies

The unilobular lungs were embedded in paraffin and 4 μm-thick histological sections were obtained. They were stained with hematoxylin-eosin (H&E) and evaluated under bright field microscopy using a Nikon Eclipse Ti microscope. The images were evaluated using the ImageJ software (ImageJ 1.40 g; W. Rasband, NIH, USA). The lung injury score (LIS) was quantified by a two blinded investigator using Table 1. The LIS was obtained by the sum of each of the five independent variables (hemorrhage, peribronchial infiltration, interstitial edema, pneumocyte hyperplasia, and intra-alveolar infiltration) and was normalized to the number of fields evaluated. The resulting injury score was a value between zero and 10 (both inclusive).

Immunofluorescence staining for surfactant C (SPC) was performed to detect ATII cells in the lung parenchyma. Deparaffinized sections were rehydrated, heated at 60 °C for 20 min in antigen retrieval buffer with 0.1 M sodium citrate and 0.1% Triton X-100, rinsed with PBS, and blocked with a solution of 3% FBS and 1% BSA for 1 h at room temperature. Next, the sections were incubated with the primary antibody SPC (Santa Cruz, USA, ref sc-13979, rabbit, 1:100) ON at 4 °C, washed with PBS, incubated with a secondary anti-rabbit antibody (Santa Cruz, USA, ref. sc3917. TR, 1:200) for 2 h at room temperature, washed with PBS, and mounted with Fluoromount Aqueous Mounting Medium (Sigma, St Louis, MO, USA). An evaluation was performed using a confocal microscope (Leica DMi8) at 630× magnification. SPC is labeled in red (Texas red).

The detection of fragmented DNA in situ was performed using a TUNEL (Terminal deoxynucleotidyl transferase dUTP nick end labeling) fluorescent assay following the manufacturer’s protocol (Roche Applied Science, Barcelona, Spain). Sections were mounted with Fluoromount Aqueous Mounting Medium (Sigma, St Louis, MO, USA), and the detection of positive cells for TUNEL in green (FITC) was performed using a confocal microscope (Leica DMi8) at 630× magnification.

### 2.9. Protein Extraction and Quantification from Lung Homogenates

Protein was extracted from the lung tissue using a lysis buffer containing 1 mM sodium orthovanadate, protease inhibitor cocktail tablets (1 tablet for 250 mg of lung tissue) (Roche; Mannheim, Germany), 0.5% Triton X-100, 150 mM of NaCl, 15 mM of Tris, 1 mM of CaCl2, and 50 mM of MgCl2 (pH 7.4) with a hand-held homogenizer. After 30 min at 4 °C incubation, the homogenates were centrifuged at 10,000× *g* at 4 °C for 20 min and then filtered with 0.45 µm Nanosep filters (Pall^®^ Life Sciences; Madrid, Spain). The total protein concentration in the lung homogenates was measured by the bicinchoninic acid method (BCA, Pierce; Thermo Scientific; Rockford, IL, USA). The cytokines were measured in lung homogenates using a Procarta rat cytokine kit (Affymetrix Inc.; Santa Clara, CA, USA) in a multiplex magnetic bead immunoassay (Luminex; Rafer, Spain) according to the manufacturer’s instructions. The amount of chemokines is expressed and corrected by the µg of protein measured at the whole lysate).

### 2.10. Statistical Analysis of Results

GraphPad-Prism7 software (GraphPad Software, Inc., La Jolla, CA, USA) was used for statistical analysis. The data were tested for Gaussian distribution applying by the D’Agostino–Pearson omnibus or Shapiro–Wilk normality testing. After we tested the data for normal distribution, we performed a one-way analysis of variance (one-way-ANOVA), followed by appropriate post-hoc tests, including the Newman–Keuls test when the differences were significant (GraphPad Software Inc., San Diego, CA, USA). A *p*-value < 0.05 was considered significant. The results of the quantitative variables were expressed as the mean ±SEM.

## 3. Results

### 3.1. Effect of Both Bell Therapies in Body and Lung Weight and Bronchoalveolar Lavage Analysis

**Figure 2:**
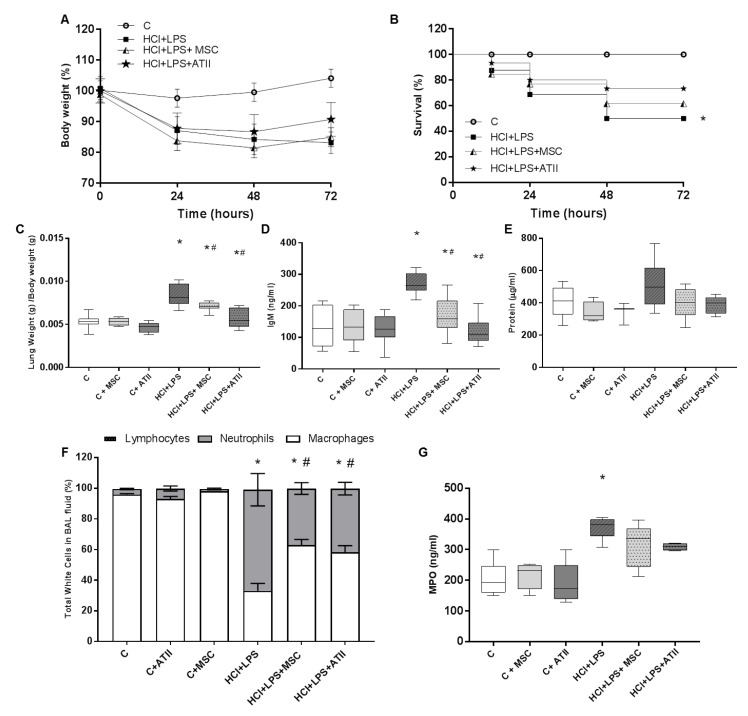
Physiologic parameters and analysis of the bronchoalveolar lavage (BAL). (**A**) Body weight every 24 h, considering 100% as the starting body weight for each group. (**B**) Survival of the animals over the timeline of the experiment. (**C**) Ratio of lung weight corrected by body weight measured at the end of the experiment (grams/grams; *n* = 6–12). (**D**) Amount of IgM measured by ELISA in BAL fluid at the endpoint as a representation of the permeability (*n* = 6–8). (**E**) Protein concentration in µg/mL in the BAL fluid at the endpoint (*n* = 6–8). (**F**) Percentage of neutrophils, macrophages, and lymphocytes in the recovered BAL fluid performed in the unilobilar lung at the endpoint. Lymphocytes are less than 1% in all groups and are almost not visible (*n* = 5–7 per group). (**G**) Myeloperoxidase (MPO) measured by ELISA in the BAL fluid (*n* = 4–6). Data are representative from 3 independent experiments (mean ± SEM; each point represents one animal). ANOVA followed by a Newman–Keuls multiple-comparison test was used to evaluate the significant differences * *p* < 0.01 vs. the corresponding control group (no differences among the three controls groups was observed in any parameter evaluated); # *p* < 0.01 vs. the HCl + LPS group.

Both cell types stabilized the body weight of injured animals at 48 h, which suggests the recovery of the animals. The injured animals instilled with HCL and LPS kept losing weight between 48 h and 72 h (Figure 2A). All the control groups gained some grams over the three days of the experiment; the three control groups are plotted together to facilitate data interpretation (Figure 2A). ALI caused the death of 50% of the rats, and survival was improved slightly by both cell therapies; no differences were observed between the treatments (Figure 2B). ATII cell and MSCs administration significantly reduced the ratio of lung weight/body weight (signal of lung damage), which was significantly increased in the injured and non-treated animals. Both cell therapies reduced the lung weight/body weight to the control values (Figure 2C). Lung permeability is one of the hallmarks of ALI, and our ALI-animal model presented a significant increase in the total protein and IgM (a big protein used to measure permeability) on the BAL (Figure 2D,E). Both cell therapies were able to significantly reduce the amount of IgM on the BAL and moderately decrease the total protein concentration, suggesting a reduction in permeability with less alveolar epithelial layer impairment (Figure 2D,E). A cell analysis was performed to evaluate the proportion of neutrophils, lymphocytes, and macrophages on the BAL. The HCL + LPS group showed a noteworthy increase in the proportion of neutrophils in comparison to the other white cells analyzed that was significantly reverted after the ATII or MSC treatment (Figure 2F). No changes were observed in the total percentage of lymphocytes in any group. Additionally, we measured myeloperoxidase (MPO) as an index of neutrophil activity and an indirect indicator of lung injury. The MPO results are consistent with the number of neutrophils in the BAL, reinforcing our observations that both cell therapies reduce neutrophil infiltration and activation (Figure 2G). 

### 3.2. MSCs and ATII Cells Diminished Inflammation after Lung Damage

**Figure 3:**
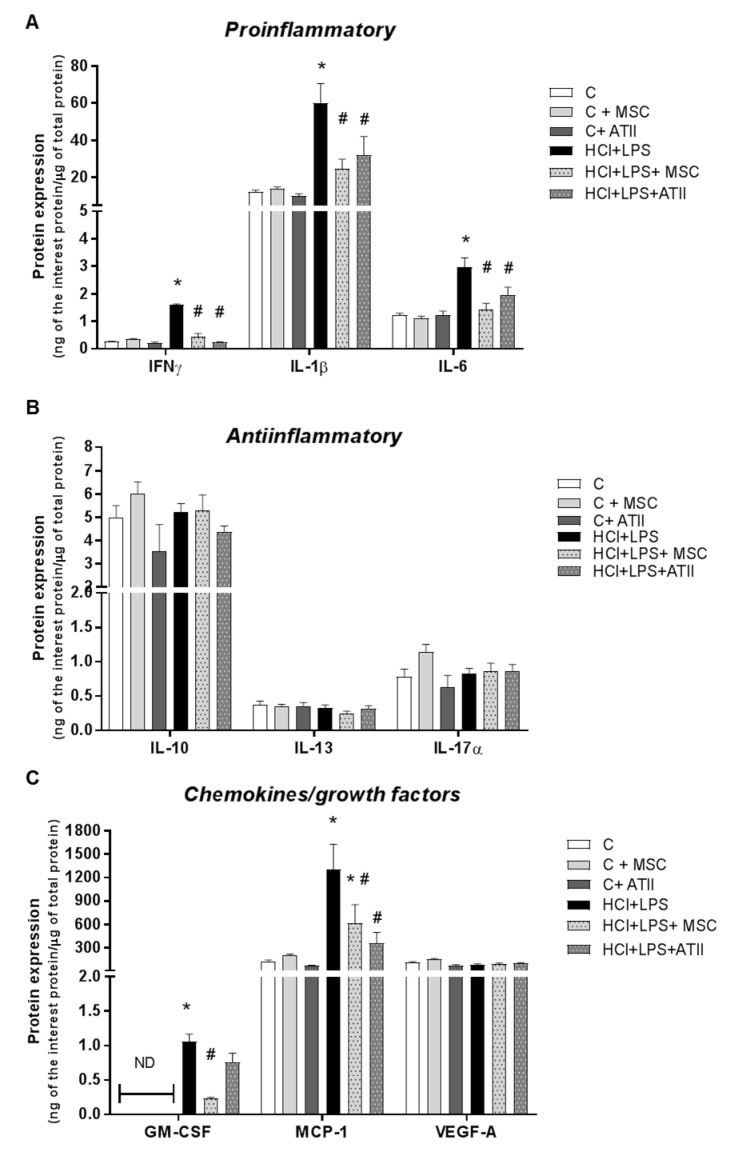
Proinflammatory and anti-inflammatory growth factors and chemoattractant mediators in the homogenates of lung tissue. (**A**) Proinflammatory mediators IFNγ, IL-1β, and IL-6. (**B**) Anti-inflammatory cytokines IL-10, IL-13, and IL-17α. (**C**) Growth factors GM-CSF and VEGF and monocyte chemoattractant chemokine MCP1. GM-CSF was not detected (ND) in any of the three control groups; for the statistical analysis, the ND value was used as the detection limit concentration. Data are representative from 2 independent experiments (*n* = 5–6 per group; mean ± SEM). One-way ANOVA followed by a Newman–Keuls multiple-comparison test was used to evaluate the significant differences * *p* < 0.01 vs. the corresponding control group (no differences among the three controls groups were observed in any parameter evaluated); # *p* < 0.01 vs. the HCl + LPS group.

We measured several proinflammatory and anti-inflammatory growth factors and chemokines related to acute lung damage in lung homogenates. Both cell therapies produced a significant decrease in the protein expression of pro-inflammatory cytokines—IFNγ, IL-1β, and IL-6—at 72 h that were significantly induced by lung injury (Figure 3A). No changes were observed between the reductions induced by both cell therapies. The anti-inflammatory markers evaluated did not show any change induced by any treatment and were also not modified for the administration of HCl and LPS to induce the ALI (Figure 3B). The protein expression of granulocyte-macrophage colony-stimulating factor (GM-CSF), responsible for neutrophil and monocyte/macrophage maturation, and monocyte chemoattractant protein 1 (MCP-1), involved in de novo monocyte recruitment, were both significantly increased in our disease ALI animal model. GM-CSF expression was not detected (ND) in the control groups because the concentrations were under the detection limit. The MSC therapy significantly reduced both factors. ATII cell therapy also reduced both factors, but only MCP-1 expression reached statistical significance. No changes were observed in the vascular endothelial growth factor (VEGF) protein expression in any groups (Figure 3C). 

### 3.3. MSC and ATII Cell Therapies Improved Lung Damage and Restore Lung Architecture

**Figure 4:**
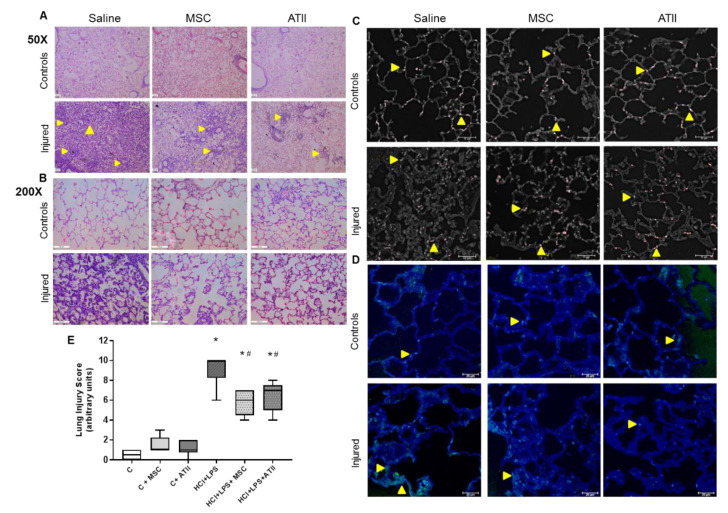
H&E histological lung sections analysis for lung injury. (**A**) Wide H&E histological sections at 50× magnification. The small yellow arrows show the multifocal lesions. (**B**) Detailed H&E histological sections at 200× magnification focus on the most representative areas to allow us to see the inflammatory cell infiltration into the alveoli and the transudate (protein/edema), shown as light pink stained areas inside the alveoli. (**C**) ATII cells stained by surfactant C (in red), and in white the autofluorescence to be able to identify the lung structures. The yellow arrows indicate SPC+ cells in the corners of the alveoli, a normal position for ATII. In the controls and transplanted groups, almost one cell per alveoli can be observed, but not in the injured and non-treated lungs. (**D**) TUNEL staining to detect apoptotic cells was performed; the pictures show “healthy” areas near an injured area for the three groups instilled with HCl + PS. In green, TUNEL+ cells are shown and they are highlighted with a small yellow arrow. In blue, the autofluorescence is shown to allow us to identify the lung structures. (**E**) Lung injury score, evaluating haemorrhage, peribronchial infiltration, interstial edema, pneumocyte hyperplasia, and intraalveolar infiltration, as described in Table 1. Data are representative from 2 independent experiments (mean ± SEM; each point represents one animal, *n* = 5–6). One-way-ANOVA followed by a Newman–Keuls multiple-comparison test was used to evaluate the significant differences * *p* < 0.01 vs. the corresponding control group; # *p* < 0.01 vs. the HCl + LPS group.

To further assess the effect of both cell therapies on the improvement of ALI, we evaluated several histological lung sections and quantified the representative hallmarks for ALI using a lung injury score (LIS) (Table 1). We screened all the sections with a small magnification to quantify the breadth of the damage (Figure 4A), then we focused on the most representative areas to evaluate the cited parameters with a higher magnification (Figure 4B). Both the cell therapies triggered a recovery of the lung structure compared to the injured and non-treated animals that were ATII cell-positive for surfactant C in the corners of the alveoli (Figure 4C). Multifocal lesions were still present in lungs transplanted with MSC or ATII cells, although, compared with non-transplanted animals, less edema, a smaller number of inflammatory cells, and considerably less hemorrhage were observed. As expected, lung tissue sections from rats with ALI showed a vast peribronchiolar and interstitial infiltration with inflammatory cells, hemorrhage, interstitial edema, and ATII cell hyperplasia in practically all the lung section. The reduction in lung lesions in both the cell-treated groups was evidenced by large areas of undamaged tissue with normal alveolar architecture (Figure 4A,B). Additionally, apoptotic cells were visualized by terminal deoxynucleotidyl transferase dUTP nick end labeling (TUNEL) staining, and overall almost no apoptotic cells were observed in the controls or transplanted animals, but a higher number of TUNEL+ cells were observed in the lungs of HCl+LPS animals (Figure 4D). A score evaluating the different parameters was performed with relevant and significant differences between the transplanted and non-transplanted animals in the ALI model (Figure 4E). Both therapies showed a comparable outcome in the analyzed parameter with comparable LIS scores, suggesting a similar tissue recovery.

## 4. Discussion

We found that MSCs and ATII cells have a similar therapeutic potential for the treatment of ALI when tested in a sustained HCl-LPS two-hit ALI model at 72 h. Both therapies were able to effectively reduce inflammation and neutrophil infiltration and recovered permeability and tissue damage. These findings are completely novel, and to our knowledge it is the first time that both therapies were compared in an identical pre-clinical ALI model using the same cell dose and route of administration. Taken together, these data offer a positive insight concerning the therapeutic and beneficial use of cell therapies for ARDS. Cell therapies have been shown to be a powerful therapeutic for several diseases, including many pulmonary diseases [2,6,14,42]. 

In this study, we evaluated the effect of cell therapies on important characteristics of human lung injury that were reproduced in our animal model [55]. We have demonstrated that both therapies have similar outcomes in the parameters evaluated. Both cellular therapies were able to partially stabilize the loss of body weight of the animals and decreased the mortality compared to the injured animals. The MSCs and ATII infusion reduced the lung permeability, which was observed as a measure of IgM and total protein in the alveolar lavage and quantified in the histological sections. The restoration of the alveolar damage depends on the equilibrium of pro-inflammatory/anti-inflammatory interactions and the modulation of many molecular pathways [43,49,56]. ARDS is characterized by an increase in IFNγ, IL-1β, and IL-6, and our pre-clinical animal ALI model shows a significant increase in the three of them [52,54,56]. The pulmonary edema fluid from ALI animals has high levels of pro-inflammatory cytokines, including interleukin IL-1β, IL-6, IL-8, and TNFα among others; protective therapies were shown to decrease IL-6 and IL-8 in ALI and ARDS [52,55,56]. In this study, MSCs and ATII reduced pro-inflammatory cytokines amounts in lung tissue, suggesting an improvement in lung injury damage. The reduction in pro-inflammatory cytokines correlates with the decrease in neutrophil infiltration and MPO in the alveolar space and a decrease in intra-alveolar and parabronchial infiltered inflammatory cells in the histological analysis. Additionally, we observed functional ATII cells in the alveoli in all transplanted groups and overall less apoptotic cells in the lunch parenchyma. We did not observe differences between both cell therapies in the parameters evaluated, and the lung damage resolution observed in our transplanted animals and the decrease in pro-inflammatory cytokines supports our conclusion that MSC and ATII have similar therapeutic effects. 

We, among others, have published the potential use of ATII cells in lung regeneration due to their ability to differentiate to alveolar type I cells and due to their abilities to secrete surfactant, which has immune-modulatory and biomechanical functions [18,24,57]. ATII cells act as immune modulators, regulating the activation of alveolar macrophages by releasing soluble factors [19]. Many studies have been published concerning the therapeutic functions of MSCs for pulmonary diseases. Numerous pre-clinical ALI models were treated with MSCs using different doses and administration routes and reported positive outcomes; all of these studies stated different mechanisms of action of these cells [7,8,9,10,13,43,44,58,59,60,61]., ALI in experimental models has many characteristics that can benefit from both cell therapies, and treatment with these cell therapies have shown positive results so far. 

Both therapies present the potential for treating patients with ARDS, but still many key challenges and a better understanding of cellular and molecular mechanism need to be addressed. In general, obtaining and maintaining MSCs in cell culture is much easier compared to ATII [62,63]. MSCs can be isolated from several adult tissues and from fetal tissues, while ATII cells are acquired from an adult´s healthy donor lungs or can be derived in vitro from pluripotent cells [35,62]. Due to the MSC immune-privileged behavior, it is possible an autologous or allogenic treatment that avoids the use of immunosuppression in patients [16,62]. The risk of the uncontrolled proliferation of MSCs and possible migration to other tissues is mitigated by the use of ATII cells. ATII are more differentiated cells than MSCs, and their characteristics make it impossible for them to survive in other organs, except the lung. Many logistic issues need to be addressed, such as the maintenance of the cells and the dose that needs to be administered, and in order to determine an appropriate administration and maintenance schedule [36,37].

Our study highlights that different cell therapies, which have been described as targeting different cellular and molecular mechanisms, appear to have similar positive effects. This study has some limitations: we do not clarify the specific mechanisms and we did not combine the ATII and MSCs into a single administration, which might enhance the positive observed effects and underline a synergistic effect between both cells. Understanding direct cell crosstalk and the factors released from each cell will open the door to the more accurate drug targeting of a specific pathway. We used a two hit model to reproduce as much ARDS heterogeneity as possible; we are aware that we used a sterile ALI model, and a more harmful infection model such as pneumonia or a non-sterile sepsis model might benefit from the ATII or MSCs differently. A confirmatory study with a non-sterile ALI model will support our conclusions and maybe call attention to the possible differences between both cell therapies.

## 5. Conclusions

In conclusion, we demonstrated that both cell therapies are successful for the treatment of ALI, with similar beneficial results and effectiveness when we administered them using the same route and dose and when we evaluated the fallouts at the same time point. Our data suggests that both cells induce the same outcome when they are administered as a therapy for acute lung injury and, as shown in previous studies, the fact that they act on different pathways and with different mechanisms of action does not change the outcome. Increasing and combining our knowledge about the compounds secreted and pathways activated by each cell therapy, we speculate that we may be able to offer new curative options in the near future for the resolution of ARDS.

## Figures and Tables

**Table 1:** Lung injury scoring system.

Parameter	Score Per Field
Haemorrhage	0–1
Peribronchial infiltration	0–1
Interstitial edema	0–2
Pneumocyte hyperplasia	0–3
Intraalveoalr infiltration	0–3
